# Reply to Fatih Kizilyel and Bedirhan Bugra Bayici

**DOI:** 10.1093/icvts/ivag123

**Published:** 2026-04-27

**Authors:** Fleur Sampon, Ferdi Akca

**Affiliations:** Department of Cardiothoracic Surgery, Catharina Hospital, 5623 Eindhoven, The Netherlands; Department of Cardiothoracic Surgery, Catharina Hospital, 5623 Eindhoven, The Netherlands

We thank Dr Kizilyel et al^1^ for their letter regarding our recent article.^2^ In this study, we conducted a retrospective national analysis of surgical treatment for single-vessel disease of the LAD performed via median sternotomy in the Netherlands. Our findings demonstrate favourable outcomes, with low 30-day and 1-year mortality, and no significant difference between off-pump (OPCAB) and on-pump (ONCAB) surgery. Off-pump surgery was associated with a reduced need for perioperative blood transfusion, whereas on-pump surgery was associated with a lower rate of target vessel reintervention.

Dr Kizilyel raises important concerns, noting that comparisons between OPCAB and ONCAB should be interpreted with caution, as the selection of the treatment strategy is not sufficiently characterized. We fully agree with this observation. Numerous factors influence the choice between different revascularization techniques, and propensity score matching of baseline characteristics alone cannot account for all nuances. In clinical practice, the Heart Team^3^ determines the most appropriate approach for each individual patient, whether this involves PCI, coronary bypass surgery, minimally invasive techniques, or hybrid strategies (combining PCI with minimally invasive surgery).^4^ Furthermore, the final surgical approach (on-pump, off-pump, thoracotomy/sternotomy) deemed most suitable by the operating surgeon based on the clinical judgement and experience is crucial. Within our national database, these additional factors are not captured, and therefore we are unable to adjust for these nuances in our analyses.

Furthermore, Dr Kizilyel comments on our concept of benchmarking the LIMA-to-LAD sternotomy group for minimally invasive techniques. In the Netherlands, minimally invasive coronary surgery is an emerging approach, with a steadily increasing number of cases in recent years. We agree that a relevant comparison extends beyond OPCAB versus ONCAB and should also include sternotomy versus minimally invasive strategies. Such comparisons have demonstrated favourable outcomes in our previous single-centre study.^5^^,^^6^

We are currently conducting a subsequent study from the national database of the Netherlands Heart Registration (NHR), which will analyse the evolution and outcomes of minimally invasive surgical revascularization of the LAD. After finalizing our study, we hope to share the results in this journal soon.

## Data Availability

The data underlying this article will be shared on reasonable request to the corresponding author.
